# Food and Environmental Virology: Use of Passive Sampling to Characterize the Presence of SARS-CoV-2 and Other Viruses in Wastewater

**DOI:** 10.1007/s12560-023-09572-1

**Published:** 2023-12-20

**Authors:** Michael Geissler, Robin Mayer, Björn Helm, Roger Dumke

**Affiliations:** 1 Institute of Medical Microbiology and Virology, University Hospital Carl Gustav Carus, Technische Universität Dresden, Dresden, Germany; 2 Institute of Urban and Industrial Water Management, Technische Universität Dresden, Dresden, Germany

**Keywords:** Wastewater, Monitoring, Virus detection, Passive sampling, Wastewater-based epidemiology, SARS-CoV-2

## Abstract

Fecal shedding of SARS-CoV-2 leads to a renaissance of wastewater-based epidemiology (WBE) as additional tool to follow epidemiological trends in the catchment of treatment plants. As alternative to the most commonly used composite samples in surveillance programs, passive sampling is increasingly studied. However, the many sorbent materials in different reports hamper the comparison of results and a standardization of the approach is necessary. Here, we compared different cost-effective sorption materials (cheesecloths, gauze swabs, electronegative filters, glass wool, and tampons) in torpedo-style housings with composite samples. Despite a remarkable variability of the concentration of SARS-CoV-2-specific gene copies, analysis of parallel-deposited passive samplers in the sewer demonstrated highest rate of positive samples and highest number of copies by using cheesecloths. Using this sorption material, monitoring of wastewater of three small catchments in the City of Dresden resulted in a rate of positive samples of 50% in comparison with composite samples (98%). During the investigation period, incidence of reported cases of SARS-CoV-2 in the catchments ranged between 16 and 170 per 100,000 persons and showed no correlation with the measured concentrations of *E* gene in wastewater. In contrast, constantly higher numbers of gene copies in passive vs. composite samples were found for human adenovirus and crAssphage indicating strong differences of efficacy of methods concerning the species investigated. Influenza virus A and B were sporadically detected allowing no comparison of results. The study contributes to the further understanding of possibilities and limits of passive sampling approaches in WBE.

**Supplementary Information:**

The online version contains supplementary material available at 10.1007/s12560-023-09572-1.

## Introduction

From the beginning of the year 2020, the rapid spread of infections by SARS-CoV-2 resulted in an unprecedented challenge for clinical capacities and established public health systems worldwide. Primarily a disease of the human respiratory tract, many investigations confirmed that coronavirus disease 2019 (COVID-19) is a systemic infection that might also affect different extra-pulmonary organ systems with multi-facetted manifestations (Kartsonaki et al., 2023). Among these, SARS-CoV-2 can cause gastrointestinal symptoms (vomiting, nausea, abdominal pain, and diarrhea) and viruses were shed in feces from the week of infection up to months after diagnosis in at least 50% of infected patients (Natarajan et al., 2022; Zhou et al., 2022). The high load of excreted viruses of approximately 10^8^ copies/g feces (Lescure et al., 2020; Pan et al., 2020) and the persistence of the nucleic acid in the aquatic environment (Atoui et al., 2023) result in the presence of measurable amounts of specific RNA in raw wastewater. Depending on local and temporal conditions, concentration of SARS-CoV-2 RNA can be associated with numbers of reported cases in the catchment area of a wastewater treatment plant (WWTP; Ciannella et al., 2023). These findings trigger a significant development of wastewater epidemiology (WBE) with numerous studies dealing with methodic aspects of virus detection in wastewater, correlation of WBE data to reported clinical cases, and optimization efforts to adjust results (Barcellos et al., 2023). Wastewater-based investigations are relatively cost-effective and have great practical advantages in comparison to clinical data: independence from number and kind of testing regime in the population as well as coverage of persons with asymptomatic or subclinical manifestations of infection. In consequence, WBE is a helpful additional tool for evaluation of the epidemiological situation in the population served by a WWTP supporting timely measurements of public health authorities.

Usually, wastewater for virological investigations were taken as composite samples allowing an integrative sampling during the period of interest (mostly 24 h). However, autosamplers are expensive and their application can be difficult under specific conditions (e.g., small sewers with low and intermitted wastewater flows, lack of electrical supply). As an alternative to composite samples, different studies have used passive sampling, which can be defined as continuous contact of a sorbent material able to concentrate viruses with the wastewater (Bivins et al., 2022). Different studies confirmed the usefulness of passive samplers for building-level investigations like in colleges or universities (Acer et al., 2022; Bivins et al., 2022; Corchis-Scott et al., 2021; Jain et al., 2022; Liu et al., 2022; Mangwana et al., 2022; Pico-Tomas et al., 2023; Wang et al., 2022), for sampling of smaller sub-catchments (Li et al., 2022; West et al., 2023), in cases with low concentrations of SARS-CoV-2 RNA (Schang et al., 2021), and in surveillance programs (Kitajima et al., 2022; Murni et al., 2022), respectively. Despite the principal suitability of the method and the agreement or even superiority of data with those obtained by grab or composite samples (Breulmann et al., 2023; Cha et al., 2023; Rafiee et al., 2021; Schang et al., 2021), the use of various sorbent materials and differences in their further processing in the laboratory make the comparison of studies difficult. Especially, the selection of the appropriate material for passive sampling remains a subject of research. Meanwhile, a broad spectrum of materials including polyethylene plastic strips (Breulmann et al., 2023), commercially available tampons (Acer et al., 2022; Bivins et al., 2022; Corchis-Scott et al., 2021; Kevill et al., 2022; West et al., 2023), cotton gauze, often described as Moore swabs (Kevill et al., 2022; Liu et al., 2022; Mangwana et al., 2022; Rafiee et al., 2021; Wang et al., 2022), cheesecloths (Hayes et al., 2021), cellulose nitrate or cellulose ester membranes (Li et al., 2022; Pico-Tomas et al., 2023) in different housings were used. Only few studies compared the efficacy of materials (Habtewold et al., 2022; Hayes et al., 2021; Kevill et al., 2022; Schang et al., 2021; Vincent-Hubert et al., 2022; Wilson et al., 2022), combined different materials (Jain et al., 2022), included more than one virus species in the wastewater monitoring with passive sampling (Hayes et al., 2023) or presented optimized materials for passive sampling like activated carbon (Hayes et al., 2022).

In the present study, the passive sampling approach was used for the detection of SARS-CoV-2 to characterize wastewater from small catchment areas in an urban environment. Special attention was paid to the selection of relatively inexpensive sorbent materials, which can be applied under conditions of limited resources. Data of passive sampling were compared with results of parallel-deposited composite samples to evaluate the practical usefulness of passive sampling. In addition, viruses representing frequently occurring human pathogenic enteric species (human adenovirus), stool-shedded viruses with pandemic potential (influenza virus A and B) as well as a commonly used indicator of fecal pollution (crAssphage) were included to proof the suitability of the method for an extended virological monitoring of wastewater.

## Materials and Methods

### Characterization of Catchment Areas

**Fig. 1:**
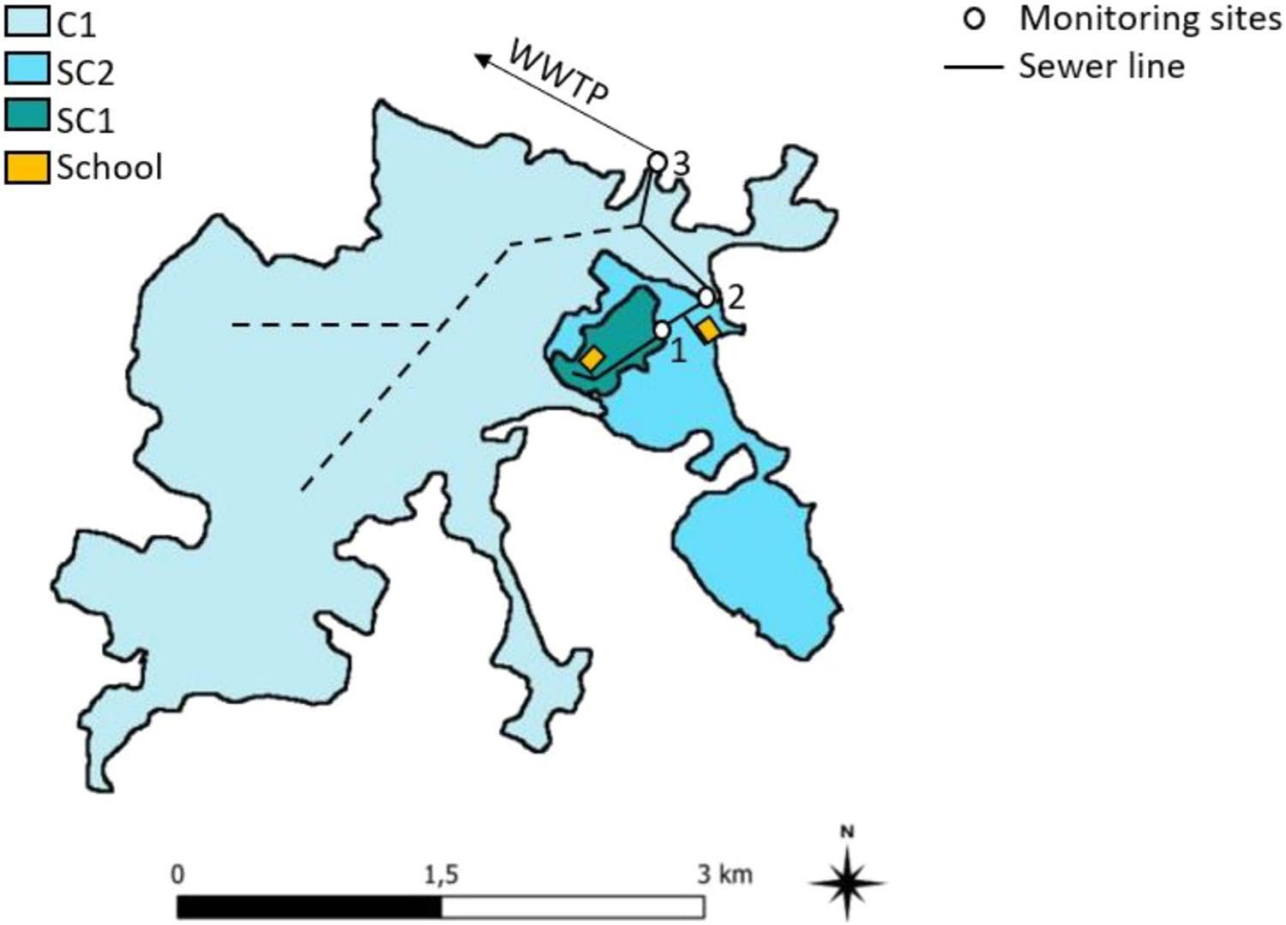
Geospatial topology of observed catchments C1, SC1, and SC2, with corresponding monitoring sites 1–3 (circles) and trunk sewer line (black line). Dotted line simulates sewer system of C1

The observed catchments are located in the southeast of the city of Dresden, Saxony, Germany. A larger catchment area (C1) and two sub-catchments located within C1 (SC1 and SC2), whose wastewater is discharged into the larger catchment area (Fig. 1), were investigated. From SC1, wastewater and stormwater are discharged separately. This separated wastewater is discharged into the combined sewer system of SC2, and from there, together with the wastewater from SC2, into the combined sewer system of C1. The respective connected inhabitants of SC1, SC2, and C1 were determined using a geospatial map of the sewer plan as well as the latest city council population census (Landeshauptstadt Dresden, 2022; Freistaat Sachsen, 2014) and are summarized in Table 1.

**Table 1:** Information on total population of all catchments and estimated population connected to observed sewer system at monitoring site

Catchment	Total population of catchment (inhabitants)	Estimated population connected to observed sewer system (inhabitants)
SC1	6.268	3.400
SC2	7.080	6.197
C1	34.935	32.235

The investigated catchment areas were examined for facilities that could lead to an increased input of pathogens. One retirement home and one school in SC2 (capacities: 269 seniors and 189 students), one retirement home in C1 (250–500 seniors), and one school in SC1 (280 students) were found, but no clinical facilities were identified from which an increased entry of SARS-CoV-2 viral residues from infected patients could be expected.

Monitoring sites 1, 2, and 3 (MS1, MS2, and MS3) are positioned at the transition points of the investigated catchments to the next larger catchment. MS2 and MS3 are part of a long-term observation study of the Institute of Urban Water Management (TU Dresden) and maintained and calibrated weekly. MS1 has been set up for the study period. Water level and flow measurements were taken at MS1 with an installed CSM correlation sensor (Nivus, Eppingen, Germany) and at MS2 and MS3 with fixed POA correlation wedge sensors (Nivus). The mean daily discharges and sampling types of the individual measuring points can be taken from Table 2.

**Table 2:** Overview of site-specific daily discharges, used autosamplers and sampling methodology

Monitoring site	Catchment	Mean daily discharge (m^3^)	Autosampler	Sampling method
MS1	SC1	400	Nemo 1 M PP (Ori, Hille, Germany)	Time proportional
MS2	SC2	1.380	P6 Mini—Vakuum (Maxx, Rangendingen, Germany)	Time proportional
MS3	C1	3.557	TP4—Vakuum (Maxx)	Flow proportional

### Sampling Campaign

In the period from July 6, 2022 to Dec 21, 2022, a total of 30 sampling campaigns were carried out in the study area. The first 10 campaigns were conducted twice per week exclusively at MS3 (catchments C1 + SC1 + SC2) to investigate different passive sampling materials. Corresponding autosampler composite samples and passive sampling housings were deployed per campaign. The sampling routine would start by setting the autosampler to take the first sample around 6:30 am, and at the same time the housings are submerged into the wastewater. Samples retrieval occurred the next day at the same time, when the housings are removed from wastewater.

### Enumeration of SARS-CoV-2-Positive Cases

Zip code-related number of reported cases of SARS-CoV-2-positive persons in the three catchment areas (C1, SC1, and SC2) during the sampling campaign were provided by the local public health authority. Incidence of SARS-CoV-2 infections covering the whole City of Dresden were taken from the official website of the Robert-Koch-Institute collecting the nation-wide data ((https://www.rki.de/DE/Content/InfAZ/N/Neuartiges_Coronavirus/Daten/Fallzahlen_Kum_Tab.html)).

### Virus Concentration from Composite Samples and Passive Sampling

Viruses in raw wastewater samples were enriched as described recently (Helm et al., 2023). Briefly, 50 ml aliquots of 24-h composite samples were centrifuged (3.000 g, 25 min, 4 °C), mixed with PEG 8000 and NaCl (head-over-head, 30 min, room temperature), and ultracentrifuged (12,000 g, 90 min, 4 °C), respectively. Sediments were resuspended in phosphate buffered saline (PBS) to a final volume of approximately 500 µl.

Passive sampling was performed using torpedo-styled samplers described by Schang et al. (2021). At sampling point M3 (June–September 2022, *n* = 10), suitability of different materials for passive sampling of SARS-CoV-2 was tested in five samplers filled with the following materials (one sampler per material at any date): (1) glass wool (Carl Roth, Karlsruhe, Germany; 8 µm, 5 g), (2) electronegative filter discs (Sartorius; cellulose nitrate, 0.45 µm; *n* = 6, corresponding to 832 cm^2^), (3) tampons (*n* = 2, approximately 6.7 g), (4) standard medical gauze swabs (Hartmann, Heidenheim, Germany; 5 × 5 cm, *n* = 5, appr. 2.5 g), and (5) cheesecloths (1.150 cm^2^, appr. 5.0 g), respectively. All samplers were exposed simultaneously in the sewer for 24 h. After transport to the laboratory (within one hour), samplers were opened, the material was transferred in beakers, and 50 ml PBS (pH 7.4) was added. Sample was mixed (300 rpm, 30 min, room temperature), the sorbent materials were transferred in 50 ml syringes and soaked/squeezed out three times. Eluates were centrifuged as described and viruses were concentrated by PEG precipitation of supernatants as described above. After selecting the appropriate material for passive sampling, variation of results with five samplers filled with cheesecloth and deposited in parallel at sampling point M3 (24 h) was investigated. Data of passive sampling were compared with corresponding composite samples (flow proportional). It is to be noted that direct comparison between both sampling approaches is difficult as the sewage volume passing the passive samples could not be quantified. However, we compared virus concentration in enriched composite samples with the concentration in 50 ml eluate after processing of passive samplers. Monitoring of SARS-CoV-2 with composite and passive sampling was performed at three different sampling points (24 h) as described before, whereas human adenovirus, crAssphage, influenza virus A and B were analyzed in samples from point M3 only. In all cases, housings and composite samples were transported, refrigerated to the laboratory and processed within the next 2 h.

### RNA Extraction, Virus Detection, and Quantification

RNA (SARS-CoV-2, influenza virus A and B) and DNA (human adenovirus, crAssphage) of viruses in wastewater and passive sampling concentrates (200 µl each) were prepared using RNeasy columns as recommended by the manufacturer (Qiagen, Hilden, Germany). In comparison with DNA mini prep columns (Qiagen), pre-investigations resulted in comparable recovery of DNA viruses (data not shown) allowing the use of RNeasy columns for all viruses of interest. Finally, nucleic acid preparations were treated to remove PCR inhibitors (Zymo Research, Irvine, CA, USA). RNA extraction and virus detection were strictly separated in different laboratories.

Detection of SARS-CoV-2, influenza viruses A and B, crAssphage, and human adenovirus by real-time quantitative PCR (RT-qPCR) were performed as reported (Dumke et al., 2022; Helm et al., 2023). Briefly, commercial kits (Altona, Hamburg, Germany) amplifying the *E* and *S* gene of SARS-CoV-2 (RealStar SARS-CoV-2 RT-PCR kit 1.0) as well as the matrix protein gene of influenza virus A and B (RealStar Influenza Screen & Type RT-PCR kit 4.0) were used according to the manufacturer’s recommendations. Amplifications were done in duplicate with undiluted samples in a QuantStudio5 thermocycler. Detection of human adenovirus and crAssphage (CPQ_064 primer/probe combination) was carried out in a CFX Opus 96 thermocycler (Bio-Rad, Hercules, CA, USA) as described (Dumke et al., 2022; Heim et al., 2003; Stachler et al., 2017) using the UCP probe PCR kit (human adenovirus; Qiagen) and the HotStarTaq master mix kit (crAssphage; Qiagen). Positive, negative, and extraction controls were included in any run (one replicate each). Prior to the nucleic acid extraction procedure, every wastewater sample and one extraction control (phosphate buffered saline (PBS)) were spiked with the same amount of artificial nucleic acid (internal control) provided by the RealStar SARS-CoV-2 RT-PCR kit. Samples with an increased Ct value for the internal control in wastewater concentrates in relation to the Ct value of the extraction control were interpreted as inhibited. Because the internal control was added prior to the extraction procedure, inhibitory effects were not explicitly assignable whether they specifically affected the extraction, the RT-qPCR or both steps. Virus concentrations were calculated with standard curves of commercial RNA standards of SARS-CoV-2 (Wuhan strain; Twist Bioscience, San Francisco, CA, USA) and of influenza virus A and B (AccuPlex Flu A/B and RSV verification kit; LGC Seracare, Milford, MA, USA) as well as of plasmids (pCR2.1-TOPO vector; Invitrogen, Waltham, MA, USA in TOP10F’ cells; Thermo Fisher, Waltham, MA, USA) carrying the target sequences for amplification of human adenovirus and crAssphage (Dumke et al., 2022). Samples were considered positive if amplification was positive in both replicates with Ct values ≤ 40.

### Data Analysis

**Fig. 2:**
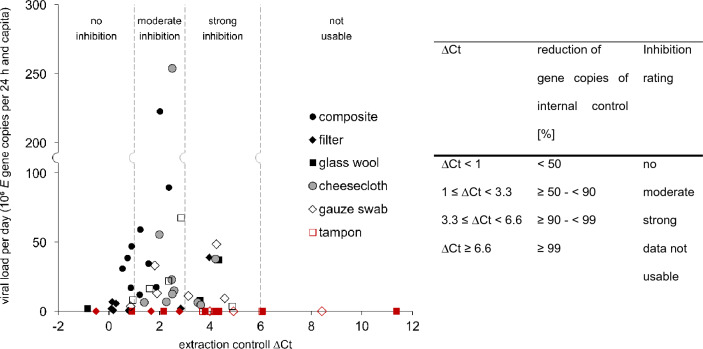
Sorbent material-related co-enrichment of PCR inhibitory compounds and their effects on *E* gene concentration. Delta Ct (∆Ct) represents the difference in the cycle threshold (Ct) of the internal control in the wastewater sample minus the Ct of the extraction control (PBS)

All virus concentrations were normalized using the flow measurement data and the population in the catchment as described recently (Langeveld et al., 2023). Results were expressed as viral load per 24 h and per capita. Despite the difficulty to quantify the volume passing the passive samplers, same procedure was performed for eluates of passive sampling as a dependence of virus concentrations and positivity rates on the wastewater flow and served population can also be expected for this approach. Mean copies of *E* gene of SARS-CoV-2 in corresponding composite and in eluates of passive samplers were compared and the standard deviation with a confidence interval of 95% (SD95) was calculated. An efficacy is given describing the percentage relation between the passive and composite sample-derived copy number. Gene concentrations in composite samples were defined as 100%. Considering the interquartile range of copies, an outlier (gauze swab sample, Aug 18, 2022) was excluded. To compare the inhibitory effect of water concentrates of both sampling approaches on RT-qPCR, the Ct values of the extraction controls were subtracted from those of the wastewater samples (∆Ct). Related to the increase in ∆Ct and to the decrease in gene concentration, different inhibiting ratings were determined (Fig. 2).

## Results and Discussion

### Efficacy of Different Sorbent Materials to Concentrate SARS-CoV-2 by Passive Sampling

To account for the complex physiochemical, hydraulic, and biological conditions of sewer systems, the ability of five different sorbent materials for the retention of SARS-CoV-2 was determined on-site at sampling point 3 (Table 3). In parallel, over the course of an exposure time of 24 h, flow-weighted composite samples were collected by autosamplers, as they are referred to provide the most representative samples (Habtewold et al., 2022). According to the difficulty to measure the flow rate through the torpedoes, a reliable determination of the SARS-CoV-2 load in wastewater after passive sampling and, in consequence, a correlation of data between both sampling approaches is difficult (Bivins et al., 2022). Different approaches are described to derive at least semi-quantitative results including parameters like volume of eluate, exposition time, flow measurement, and surface of sorption material (Acer et al., 2022; Hayes et al., 2021; Mangwana et al., 2022; Schang et al., 2021; Vincent-Hubert et al., 2022) but other authors emphasize the qualitative character of passive sampling data (Jain et al., 2022; Liu et al., 2022; Wang et al., 2022). Despite this general problem of passive samplers, we compared virus concentrations in PEG-enriched composite samples and in eluates after processing the sorption material(s) in corresponding passive samples to get a rough estimation of efficiency of both approaches to concentrate SARS-CoV-2 in wastewater. All composite samples were found positive for *E* gene (10/10) with concentrations ranged from 1.5 × 10^7^ up to 2.3 × 10^8^ copies per 24 h and capita (mean: 5.4 × 10^7^ copies per 24 h and capita, SD95: 1.5 × 10^8^ copies per 24 h and capita). For the passive sampling materials, the highest number of *E* gene copies in the eluates was obtained with cheesecloths (mean: 4.1 × 10^7^ copies per 24 h and capita, SD95: 1.8 × 10^8^ copies per 24 h and capita) and no negative sample as well (10/10). In comparison to the composite samples, an efficacy of 66.9% (SD95: 117.7%) is calculated. It must be pointed out that cheesecloth is not a laboratory grade material which should be preferred due to standardized production and composition (Hayes et al., 2021). Moore swabs, here referred as gauze swabs, are the most frequently used material and was shown to be successful for the detection of SARS CoV-2 in wastewater (Cha et al., 2023; Habtewold et al., 2022; Liu et al., 2022; Mangwana et al., 2022; Rafiee et al., 2021; Schang et al., 2021; Wang et al., 2022) . In the present study, seven out of ten gauze swab samples were positive for *E* gene of SARS-CoV-2 and the mean concentration was 1.8 × 10^7^ copies per 24 h and capita (SD95: 4.0 × 10^7^ copies per 24 h and capita) which corresponds to a mean efficiency of 57.9% (SD95: 212.2%). For these calculations, the measurements of Aug 18, 2022 were excluded and considered as a positive outlier. As glass wool was already used for concentration of different viruses from water (Ikner et al., 2012), its capability to serve as a sorbent material for SARS-CoV-2 was also determined. With a positive rate of 3/10, a mean *E* gene concentration of 1.4 × 10^7^ copies per 24 h and capita (SD95: 7.3 × 10^7^ copies per 24 h and capita), and an efficacy of 6.7% (SD95: 31.5%) in comparison with composite samples, glass wool showed a relatively low efficacy to concentrate SARS-CoV-2. Rate of SARS-CoV-2 positivity for the other sorbent materials was 70% (electronegative filter) and 50% (tampons) with mean concentrations of positive samples of 7.3 × 10^6^ (filter) and 2.1 × 10^7^ (tampons) gene copies per 24 h and capita. Both materials were used in different studies (Corchis-Scott et al., 2021; Wilson et al., 2021; Acer et al., 2022; Jain et al., 2022; Kevill et al., 2022; Li et al., 2022; West et al., 2023) but are not compared to cheesecloths. Only the report by Hayes et al. (2021) confirmed the suitability of cheesecloth as sorbent material. Additionally, for concentration of eluates, different methods are used like skimmed milk flocculation (Liu et al., 2022; Wang et al., 2022) or filtration (Corchis-Scott et al., 2021; Habtewold et al., 2022). Here, precipitation with PEG was performed. The procedure resulted in relatively high recovery rates (Dumke et al., 2021) and was demonstrated to be efficient in passive sampling studies (Cha et al., 2023; Rafiee et al., 2021).

**Table 3:** Comparison of different sorbent materials for passive sampling of SARS-CoV-2 (sampling point 3, exposure time: 24 h). *E* gene copies per 24 h and capita

Date	24-h composite sample	Filter	Glass wool	Cheesecloth	Gauze swab	Tampon
Jul 7, 2022	1.1 × 10^7^	6.3 × 10^6^	neg.^a^	1.3 × 10^7^	3.0 × 10^7^	2.0 × 10^7^
Jul 14, 2022	8.1 × 10^7^	3.5 × 10^7^	3.4 × 10^7^	1.1 × 10^7^	3.3 × 10^6^	6.1 × 10^7^
Jul 20, 2022	3.5 × 10^7^	5.1 × 10^6^	7.2 × 10^6^	5.0 × 10^7^	4.4 × 10^7^	3 × 10^6^
Jul 21, 2022	3.1 × 10^7^	1.8 × 10^6^	neg	3.4 × 10^7^	8.5 × 10^6^	neg
Jul 27, 2022	2.8 × 10^7^	neg	neg	2.1 × 10^7^	neg	neg
Jul 28, 2022	1.5 × 10^7^	5.0 × 10^5^	neg	5.8 × 10^6^	1.0 × 10^7^	neg
Aug 17, 2022	5.3 × 10^7^	8.0 × 10^5^	neg	6.3 × 10^6^	1.2 × 10^7^	1.5 × 10^7^
Aug 18, 2022	4.2 × 10^7^	1.7 × 10^6^	1.9 × 10^6^	5.8 × 10^6^	6.1 × 10^8^	7.4 × 10^6^
Aug 31, 2022	1.6 × 10^7^	neg	neg	4.2 × 10^6^	neg	neg
Sep 14, 2022	2.3 × 10^8^	neg	neg	2.6 × 10^8^	neg	neg

^a^neg.—none detected

Several reports demonstrated that negative results or reduced copy numbers of passive samples might occur due to a co-enrichment of PCR inhibitory compounds beside the target substance (Cha et al., 2023; Habtewold et al., 2022; Schang et al., 2021). To determine the effect of inhibitory substances in the present study, an extraction control was co-processed during the extraction of nucleic acids and Ct values of the internal control in extraction control and in wastewater samples were compared (∆Ct). The effect of ∆Ct on the number of *E* gene copies in samples enriched with different sorbent materials is summarized in Fig. 2. Composite samples with a mean ∆Ct of 1.33 (SD95: 1.37) were affected by a moderate inhibition. For filters, the mean ∆Ct of 1.22 (SD95: 3.41) was correlated with the lowest mean copy number of *E* gene (7.3 × 10^6^ copies per 24 h and capita, SD95: 3.1 × 10^7^ copies per 24 h and capita) for all tested materials and therefore could be interpreted as the result of an overall insufficient adsorption rate. Although the mean ∆Ct of 2.71 (SD95: 1.90) for the cheesecloth samples was nearly twice as high as for the composite samples, the difference could not found to be significant (*t*(16) = −1.858, *p* = 0.08). The low positive rate (3/10) and copy number of *E* genes (1.4 × 10^7^ copies per 24 h and capita) of glass wool correlated with a high mean ∆Ct of 4.00 (SD95: 7.37), which results in the loss of 90% up to 99% of gene copies in these samples. A mean copy number of *E* gene absorbed by gauze swabs and tampons of 1.8 × 10^7^ copies per 24 h and capita (SD95: 4.0 × 10^7^ copies per 24 h and capita) and 2.1 × 10^7^ copies per 24 h and capita (SD95: 6.4 × 10^7^ copies per 24 h and capita) was determined. However, analysis of both materials was also affected by PCR inhibition, characterized by an increased mean ∆Ct of 3.48 (SD95: 5.19) and 3.50 (SD95: 3.52) and a decreased rate of positive samples of 70% and 50%, respectively. Conclusively, PCR inhibition seems an important aspect of passive sampling and should be considered. For broad application of passive samplers it is necessary to optimize the sampling procedure to ensure the detection of the maximum number of viruses in combination with minimal amount of co-adsorbed inhibitory compounds. Furthermore, from a practical point of view, it has to be noted that processing the sorption material of passive sampling in the laboratory needs more time in comparison to composite samples.

### Replicability of Passive Sampling-Based Measurements of SARS-CoV-2 Using Cheesecloths

To determine the replicability and variability of passive sampling measurements, five cheesecloth-loaded passive samplers were simultaneously deployed in the wastewater stream of monitoring site 3 (Table 4). As a reference, composite samples were taken in parallel. Composite samples were found to be in a range of 150 copies per ml up to 392 copies per ml showing in three out of four samples a lower virus concentration of wastewater from the catchments C1, SC1, and SC2 in comparison with the inlet of the central wastewater treatment plant of City of Dresden at the same day. Lowest concentration of *E* gene copies in composite samples (150 copies per ml) correlates with negative results in all five corresponding passive samples, whereas number of positive amplifications after enrichment with cheesecloths ranged between 1/5 and 5/5 for the remaining sampling events. With one sampling positive for all five passive samplers, a coefficient of variation of 24.6% of gene concentration was calculated. Virus concentration in composite and passive samples were measured at 243 copies per ml and a mean of 59 copies per ml (SD95: 188 copies per ml) with passive samples in a range from 14 to 177 copies per ml. In contrast, at one sampling event (Dec 7, 2022) with a composite sample showing a relatively high SARS-CoV-2 concentration (392 gene copies per ml), a low positive rate of 1/5 (150 copies per ml in the positive sample) was found using the passive samplers. Overall, investigation of five passive samplers deposited in parallel in the sewer resulted in a great variability not only regarding the virus concentrations measured as well as the positivity rate of SARS-CoV-2 detections.

**Table 4:** Variation of SARS-CoV-2 concentrations in composite samples (C) and in five corresponding passive samples using cheesecloths (P1-5, sampling point 3, exposure time: 24 h)

Date	*E* gene copies/ml						
	C WWTP^a^	C3	P1	P2	P3	P4	P5
Nov 29, 2022	601	292	302	neg.^b^	neg	68	neg
Dec 7, 2022	199	392	neg	neg	150	neg	neg
Dec 20, 2022	664	243	49	14	177	17	36
Dec 21, 2022	393	150	neg	neg	neg	neg	neg

^a^WWTP—central wastewater treatment plant of City of Dresden

^b^neg.—none detected

Even for positive composite samples in a moderate to high concentration range, the corresponding passive samplers were affected by outages (temporarily ragged housing) and therefore limited positive rates, that concomitant with a high variance in copy number. In consequence, use of passive samplers for the collection of quantifiable data is debatable. Further research in the determination of particularly important parameters (accumulation ratio, duration until saturation) and wastewater flow characteristics should lead to an improved sampling that maximizes the number of accumulated viruses with minimal variations. Accompanied with an increased sensitivity and replicability, qualitative measurements with passive samplers could be considered as an acceptable option for an easy-to-use early detection system. The accumulation ratio for three different sorbent materials (electronegative membranes, cotton buds, gauze) over independent time periods was determined by Habtewold et al. (2022). Exemplarily, gauze was defined by a variable, non-linear adsorption rate for SARS-CoV-2 with a saturation after four hours that equals the viral load of membranes and cotton buds after 48 h. As stated by Bivins et al. (2022), wastewater flows in collection system are often characterized by pulse inlets. Thus, information about the diurnal wastewater flow profile should be used to determine peak times in volume flow rate. At the central WWTP in Dresden, 12 × 2-h composite samples (*n* = 4) were taken between 6 am and 6 pm. The diurnal profile was characterized by an increased SARS-CoV-2 concentration between 8 am and 4 pm with a mean concentration of 1.3 × 10^6^ copies per ml. The mean concentration of the corresponding 24-h composite samples was only half as much with 0.7 × 10^6^ copies per ml (data not shown). Sampling at wastewater flow peak times would improve the chance of virus presence at higher concentrations, but also reduce the sampling time and therefore minimize the separation rate of already-adsorbed viruses. Such an improved procedure could be considered as an important step towards a stable passive sampler-based wastewater monitoring.

### Monitoring of SARS-CoV-2 with Passive Samplers Containing Cheesecloths

During Sept 27, 2022 and Dec 21, 2022, corresponding 24-h composite samples and cheesecloth-based passive samples were taken at three different sampling sites (Fig. 1, table S1). Summarizing all samples from site 1–3 (*n* = 49) that were positive for both sampling methods (*n* = 24), virus concentration in 83% of samples measured after composite sampling was higher than for passive sampling with a mean factor of 8.5 (SD95: 23.0). Between each sampling method, the virus concentrations calculated at monitoring sites 1 and 2 were in a similar range. Based on composite sampling, 2.0 × 10^7^ copies per 24 h and capita (SD95: 3.2 × 10^7^ copies per 24 h and capita) at site 1 and 8.9 × 10^7^ copies per 24 h and capita at site 2 (SD95: 1.3 × 10^8^ copies per 24 h and capita) were detected. Similar results among corresponding passive sampling were observed resulting in mean virus concentrations of 7.1 × 10^6^ copies per 24 h and capita at site 1 (SD95: 1.3 × 10^7^ copies per 24 h and capita) and 3.1 × 10^7^ copies per 24 h and capita at site 2 (SD95: 9.1 × 10^7^ copies per 24 h and capita). In comparison to the wastewater of catchments SC1 and SC2, a higher number of gene copies was registered at monitoring site 3. Mean virus concentrations of 5.7 × 10^7^ copies per 24 h and capita for composite samples (SD95: 7.1 × 10^7^ copies per 24 h and capita) and 2.6 × 10^7^ copies per 24 h and capita (SD95: 6.1 × 10^7^ copies per 24 h and capita) for passive samples were determined. Tests to establish a correlation of the number of viruses retained by composite and passive samples including all samples from sites 1–3 (n = 49) were negative, confirmed by a low determination coefficient of *R*^2^ = 0.00768.

The positive rates of composite and passive samples for all samples of sites 1–3 (*n* = 49) were 98.0% and 50.0%, respectively. For composite sampling, no outages occurred at sites 2 and 3 with positives rates of 100% (*n* = 18) and of 94.4% (*n* = 18) at site 1 with only one negative sample. With positive rates of 57.9% (*n* = 19), 56.3% (*n* = 16), and 36.8% (*n *= 19) for passive sampling at sites 1–3, relatively slight site-related deviations were observed. Regarding the positive rate, composite sampling showed great advantages in comparison to use of passive samples. Lower numbers of detected gene copies or negative measurements were mainly observed for passive samples. For the majority of these samples, a higher Ct value of the internal control in comparison to the extraction control was measured (data not shown). Further experiments should evaluate the proportion of adsorbed virus particles and inhibitory compounds in relation of the sampling duration and the local characteristics of wastewater.

**Fig. 3:**
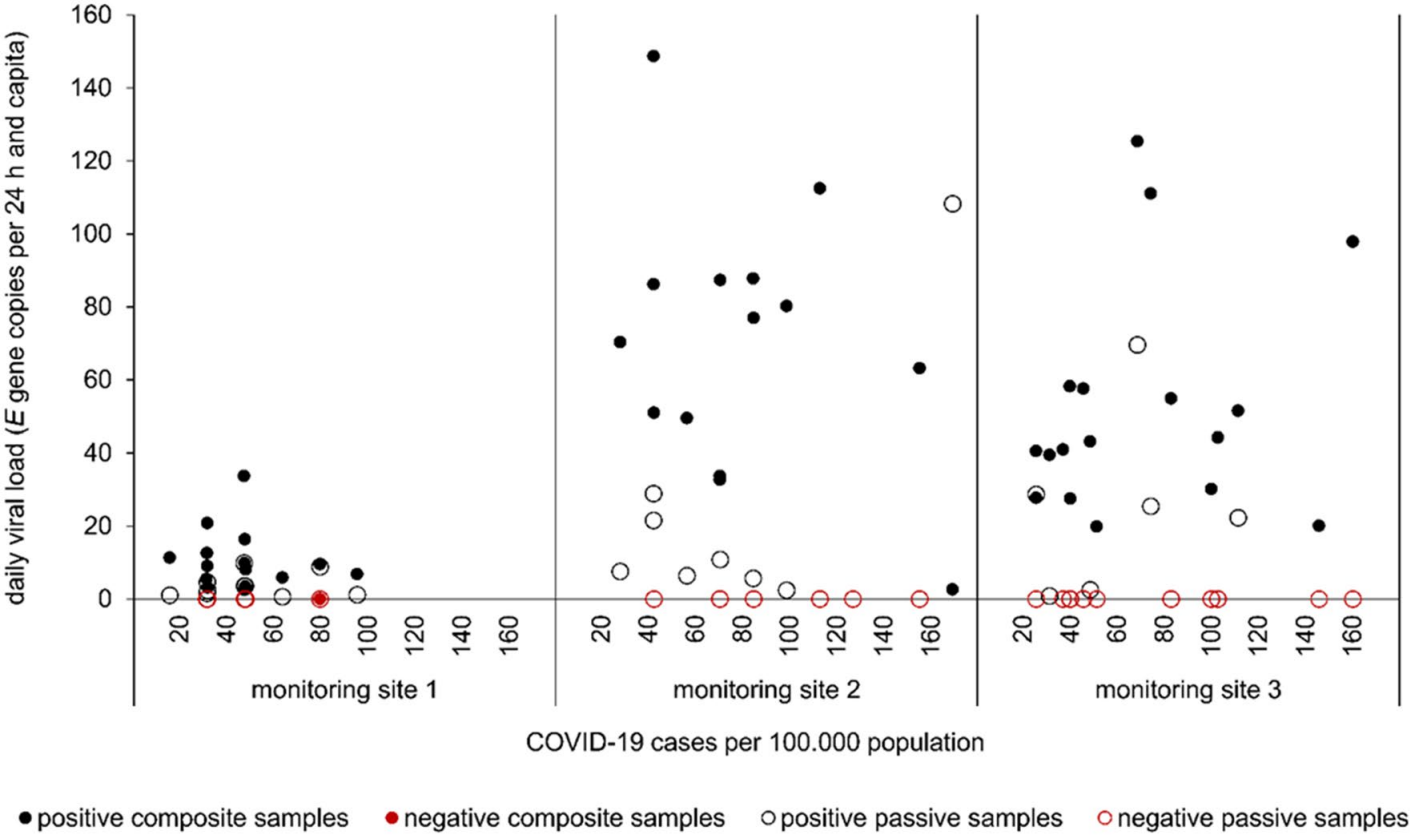
Composite and passive sampling-based monitoring of SARS-CoV-2 in catchments C1 (site 3), SC1 (site 1), and SC2 (site 2) related to the reported COVID-19 cases per 100.000 population

For monitoring site 1–3, the number of daily registered COVID-19 cases for each of the corresponding catchments was collected and transferred into COVID-19 cases per 100,000 population (Fig. 3). Incidences in catchments SC2 and C1 were in a similar range of 28.2—169.5 (mean: 79.8) and 25.8—160.3 (mean: 67.7). Slightly lower incidences were observed at the largest catchment C1 starting at 16.0 up to 95.7 with a mean of 48.8 per 100,000. During the sampling campaign, in the whole Dresden area incidences in a range of 84.5—604.7 (mean: 275.8) were reported. For correlation analysis between catchment-related incidences and the virus concentration at the corresponding sample point, negative samples were interpreted as a concentration of 0 copies per 24 h and capita. Neither with composite nor passive sampler-based sampling, a correlation was found. Determination coefficients *R*^2^ for the individual catchments ranged from 0.00006 up to 0.15606. Based on an extended data set of pooled samples from site 1–3 (*n* = 48), only low determination coefficients of *R*^2^ = 0.08328 for composite samples and *R*^2^ = 0.07015 for passive samples were calculated. An underestimation of the official COVID-19 case numbers is probably causal for the absence of a correlation. With the Saxon Corona Protection Ordinance from March 31, 2022 coming to effect, the need to proof a negative COVID-19 test result to participate in public life was disestablished. Throughout Germany, the number of weekly registered tests decreased by 78.8% (1.969781 to 771.107 tests per week) until start of the passive sampling campaign. At the end of the sampling campaign at calendar week 51/22, the number of weekly registered tests dropped by additional 45.8% (418,231 tests per week; https://www.rki.de/DE/Content/InfAZ/N/Neuartiges_Coronavirus/Daten/Testzahlen-gesamt.html). In consequence, reported cases of SARS-CoV-2 infections during the sampling campaign will only roughly reflect the real epidemiological situation in the population. For the evaluation of data, this aspect should be taken into account. Used procedure to detect SARS-CoV-2 in composite samples allows positive results up to an incidence of at least around 20 cases per 100,000 persons (one negative sample at 80 cases per 100,000, Fig. 3). In contrast, different results of passive samples are positive in the same range but RT-qPCR remained negative in many samples up to the maximal incidence of 160 cases per 100,000.

### Detection of Further Wastewater-Carried Viruses in Composite and Passive Samples

**Fig. 4:**
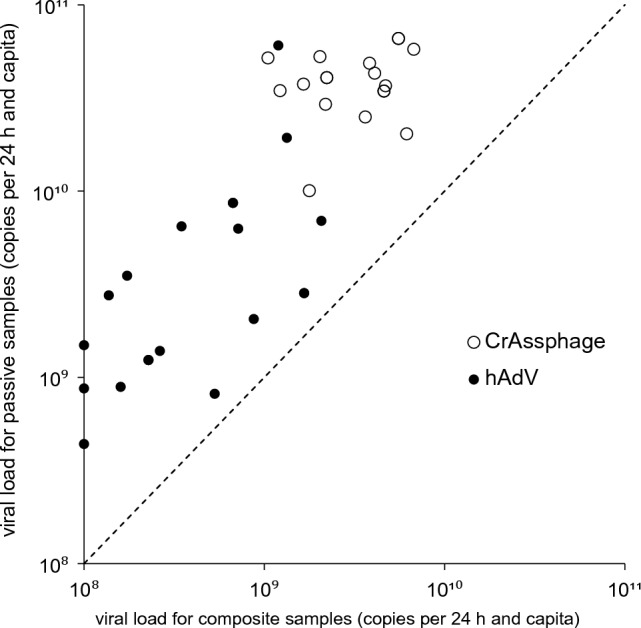
Comparison of concentrations of gene copies of crAssphage and human adenovirus (hAdV) in composite and passive samples (sampling point 3)

To expand the panel of surveyed human fecally shedding viruses, the wastewater at monitoring site 3 was also screened for crAssphage, human adenovirus, influenza virus A and B, respectively (Fig. 4, table S2). CrAssphages were used as an indicator for human-fecal contamination in many studies (Sabar et al., 2022). Adenoviruses are among the “classical” enteric viruses occurring in high concentrations and without seasonal trend in wastewaters globally (Haramoto et al., 2018) and were also proposed as an indicator of virological quality of waters (Rames et al., 2016). Influenza viruses were excreted with feces of infected persons and increasingly studied in wastewater to establish an early warning system by WBE.

Here, great differences between the detection of SARS-CoV-2, influenza virus A and B, crAssphage, and human adenovirus by passive sampling are demonstrated. The positive rate for crAssphage and human adenovirus was 100% for both sampling methods. However, higher virus concentrations were measured in passive samples. The mean concentration of crAssphages detected by passive sampling was 17.0 (SD95: 26.7) times larger in comparison to composite samples (mean concentration of 4.8 × 10^10^ ± SD95: 5.9 × 10^10^ copies per 24 h and capita for passive sampling and 3.6 × 10^9^ ± SD95: 4.1 × 10^9^ copies per 24 h and capita for composite sampling). A higher mean concentration for passive sampling in comparison to composite samples was also found for the indicator virus PMMoV in a study by Cha et al. (2023)*.* Using passive and composite samples for detection of human adenovirus, mean concentrations of 7.4 × 10^9^ copies per 24 h and capita (SD95: 3.1 × 10^10^ copies per 24 h and capita) and 6.2 × 10^8^copies per 24 h and capita (SD95: 1.3 × 10^9^ copies per 24 h and capita) were determined. The virus concentrations measured by both sampling methods differ by a mean factor of 12.1 (SD95:25.0). To our knowledge, efficacy of passive sampling for enrichment of adenovirus from sewers has not been studied so far. In laboratory-scale experiments, recovery rates in cotton-based passive samplers and composite samples were comparable (Kevill et al., 2022). Influenza virus A was not detectable in passive samples and with a positive rate of 23.5% (4/17) by composite sampling (mean concentration: 1.0 × 10^7^ copies per 24 h and capita, SD95: 3.2 × 10^7^ copies per 24 h and capita). Influenza virus B was detectable in higher mean concentrations of 3.7 × 10^7^ copies per 24 h and capita (SD95:1.6 × 10^8^) for passive sampling and 2.8 × 10^7^ copies per 24 h and capita (SD95: 9.7 × 10^7^ copies per 24 h and capita) for composite sampling. However, rate of positive samples was relatively low in both cases (3/17 for passive sampling and 6/17 for composite sampling). At only one date (26-October), influenza virus B qPCR was positive for both sampling methods. The results agree with data of other studies confirming the successful detection of influenza virus in wastewater and the potential of a wastewater-based surveillance to support measurements of public health authorities regarding the course of the yearly influenza epidemics. Interestingly, these studies confirmed very different specific gene concentrations and rates of positive samples in WWTP and building-level monitorings (Ahmed et al., 2023; Ando et al., 2023; Boehm et al., 2023; Dumke et al., 2022; Hayes et al., 2023; Johnson et al., 2022; Toribio-Avedillo et al., 2023; Wolken et al., 2023) suggesting regional or even local differences (e.g., rate of immunization, presence of hot spots in the catchment like hospitals, current incidence). The limited detection rate of influenza virus resulted in difficulties to correlate wastewater data with the incidence of infections. Between October and December 2023, the number of reported influenza cases in the City of Dresden (data from the investigated catchment areas are not available) ranged from 10 to 916 with highest case numbers in December (https://www.gesunde.sachsen.de/influenza). These clinical data were largely reflected by the wastewater monitoring showing first influenza virus A-positive samples in November and an increase of genome concentrations in December 2023. In contrast, pattern of influenza virus B presence in wastewater seems to be more stochastic which might be due to the clinical testing regime with a lack of differentiation between influenza virus A and B in any case, unknown dynamics of fecal excretion of viruses, and differences in the clinical course of infections by both influenza viruses.

## Conclusions

This study investigated the potential of passive sampling in capturing SARS-CoV-2 and other viruses in wastewater of small urban catchments. Our experiences suggest that cheesecloths are a cost-effective and easily accessible sorption material for passive sampling of viruses. However, regarding SARS-CoV-2, passive sampling was associated with lower rates of positive samples and decreased genome concentrations in comparison with composite samples. Thus, wastewater monitoring with these passive samplers might be helpful in cases of low-resource settings, of conditions that rule out composite samples or of particular monitoring programs (e.g., building-level surveillance, qualitative screening of waters during periods with low incidence levels). In this connection, passive sampling is a valuable tool for epidemiological investigations giving an overview of the occurrence of SARS-CoV-2 in the connected population. Further studies are necessary to demonstrate the practicability of the approach for detection of other viral families in wastewater with relevance for public health. We demonstrated that, in comparison to composite samples, the passive sampling method was very efficient for concentration of human adenoviruses and crAssphages suggesting an application to further species.

### Supplementary Information

Below is the link to the electronic supplementary material.

Supplementary file1 (DOCX 19 KB)
