# Supplementary material for: Food and Environmental Virology: Use of Passive Sampling to Characterize the Presence of SARS-CoV-2 and Other Viruses in Wastewater
Source: Food Environ Virol. 2023 Dec 20;16(1):25–37. doi: 10.1007/s12560-023-09572-1 (PMC10963477; doi:10.1007/s12560-023-09572-1)
Supplement: Supplementary file 1 — Supplementary file1 (DOCX 19 KB) [file 12560_2023_9572_MOESM1_ESM.docx]

**Supplemental material**

**Table S1** Detection of SARS-CoV-2 in 24 h composite wastewater (C) and passive samples (P, exposure time: 24 h) at sampling points 1-3. *E* gene copies per 24 h and capita.

| Date | Sampling point | | | | | | |
| --- | --- | --- | --- | --- | --- | --- | --- |
|  | C  WWTP^a^ | C1 | P1 | C2 | P2 | C3 | P3 |
| 27-Sep-2022 | 1.7 × 10^8^ | 6.2 × 10^7^ | 1.8 × 10^7^ | n.t.^b^ | 6.3 × 10^7^ | 1.2 × 10^8^ | 2.8 × 10^7^ |
| 28-Sept-2022 | 2.5 × 10^8^ | 1.8 × 10^7^ | neg.^c^ | 1.3 × 10^8^ | neg. | 1.4 × 10^8^ | 7.5 × 10^7^ |
| 4-Oct-2022 | 1.4 × 10^8^ | 1.3 × 10^7^ | 2.2 × 10^6^ | 7.2 × 10^7^ | neg. | 5.6 × 10^7^ | 2.4 × 10^7^ |
| 5-Oct-2022 | 1.6 × 10^8^ | 1.8 × 10^7^ | 1.6 × 10^7^ | 1.0 × 10^8^ | 6.5 × 10^6^ | 2.2 × 10^7^ | neg. |
| 11-Oct-2022 | 2.8 × 10^8^ | 1.0 × 10^7^ | neg. | 2.6 × 10^8^ | neg. | 1.1 × 10^8^ | neg. |
| 12-Oct-2022 | 2.3 × 10^8^ | 4.6 × 10^6^ | 6.6 × 10^6^ | 9.8 × 10^7^ | 3.3 × 10^7^ | 3.3 × 10^7^ | neg. |
| 18-Oct-2022 | 2.0 × 10^8^ | neg. | neg. | 3.0 × 10^6^ | 1.2 × 10^8^ | n.t. | 1.7 × 10^7^ |
| 19-Oct-2022 | 2.4 × 10^8^ | 1.1 × 10^7^ | 1.2 × 10^6^ | 9.2 × 10^7^ | 2.7 × 10^6^ | 5.9 × 10^7^ | neg. |
| 25-Oct-2022 | 1.2 × 10^8^ | 3.0 × 10^7^ | neg. | 5.7 × 10^7^ | 7.3 × 10^6^ | 4.8 × 10^7^ | neg. |
| 26-Oct-2022 | 1.4 × 10^8^ | 2.1 × 10^7^ | 1.9 × 10^6^ | 3.7 × 10^7^ | neg. | 4.7 × 10^7^ | 2.7 × 10^6^ |
| 8-Nov-2022 | 1.1 × 10^8^ | 3.5 × 10^7^ | neg. | 3.9 × 10^7^ | neg. | 6.3 × 10^7^ | neg. |
| 9-Nov-2022 | 1.1 × 10^8^ | 2.3 × 10^7^ | 2.8 × 10^6^ | 1.7 × 10^8^ | 2.5 × 10^7^ | 4.3 × 10^7^ | 9.0 × 10^5^ |
| 22-Nov-2022 | 1.3 × 10^8^ | n.t. | 1.0 × 10^7^ | 8.0 × 10^7^ | 8.6 × 10^6^ | 4.4 × 10^7^ | neg. |
| 23-Nov-2022 | 7.5 × 10^7^ | 6.4 × 10^6^ | 6.5 × 10^6^ | 1.0 × 10^8^ | 1.2 × 10^7^ | 3.0 × 10^7^ | neg. |
| 29-Nov-2022 | 9.7 × 10^7^ | 1.7 × 10^7^ | neg. | 5.8 × 10^7^ | neg. | 3.0 × 10^7^ | 3.1 × 10^7^ |
| 30-Nov-2022 | 6.5 × 10^7^ | 4.8 × 10^6^ | neg. | 3.8 × 10^7^ | n.t. | 4.4 × 10^7^ | neg. |
| 6-Dec-2022 | 3.0 × 10^7^ | 1.5 × 10^7^ | neg. | n.a. | neg. | n.t. | neg. |
| 7-Dec-2022 | 4.4 × 10^7^ | 3.8 × 10^7^ | 8.5 × 10^6^ | n.a. | neg. | 6.2 × 10^7^ | neg. |
| 20-Dec-2022 | 1.8 × 10^8^ | 5.7 × 10^6^ | 4.2 × 10^6^ | n.t. | neg. | n.a. | n.a. |
| 21-Dec-2022 | 8.9 × 10^7^ | n.t. | neg. | 8.8 × 10^7^ | neg. | 2.2 × 10^7^ | neg. |

^a^ WWTP – composite sample at the central wastewater treatment plant of City of Dresden

^b^ n.t. - not tested

^c^ neg. - none detected

^d^ n.a. - not applicable (failure of flow measurement)

**Table S2** Detection of crAssphage, adenovirus, influenza viruses A and B in 24 h-composite wastewater (M) and passive samples (P) at sampling point 3. Genome copies per 24 h and capita.

| Date | CrAssphage | | Human Adenovirus | | Influenza virus A | | Influenza virus B | |
| --- | --- | --- | --- | --- | --- | --- | --- | --- |
|  | C3 | P3 | C3 | P3 | C3 | P3 | C3 | P3 |
| 27-Sep-2022 | 2.2 × 10^9^ | 2.9 × 10^10^ | 1.6 × 10^8^ | 8.9 × 10^8^ | neg.^a^ | neg. | 7,4 × 10^6^ | neg. |
| 28-Sept-2022 | 4.1 × 10^9^ | 4.3 × 10^10^ | 1.7 × 10^8^ | 3.5 × 10^9^ | neg. | neg. | neg. | neg. |
| 4-Oct-2022 | 6.2 × 10^9^ | 2.0 × 10^10^ | 2.1 × 10^9^ | 6.9 × 10^9^ | neg. | neg. | 5.7 × 10^7^ | neg. |
| 5-Oct-2022 | 6.8 × 10^9^ | 1.1 × 10^11^ | 7.2 × 10^8^ | 6.3 × 10^9^ | neg. | neg. | neg. | neg. |
| 11-Oct-2022 | 6.7 × 10^9^ | 5.8 × 10^10^ | 3.5 × 10^8^ | 6.5 × 10^9^ | neg. | neg. | neg. | neg. |
| 12-Oct-2022 | 5.5 × 10^9^ | 6.6 × 10^10^ | 6.7 × 10^8^ | 8.6 × 10^9^ | neg. | neg. | 1.1 × 10^6^ | neg. |
| 18-Oct-2022 | neg. | 8.4 × 10^9^ | n.t.^b^ | 2.0 × 10^6^ | n.t. | neg. | n.t. | neg. |
| 19-Oct-2022 | 3.8 × 10^9^ | 4.9 × 10^10^ | 1.3 × 10^9^ | 1.9 × 10^10^ | neg. | neg. | neg. | neg. |
| 25-Oct-2022 | 3.6 × 10^9^ | 2.5 × 10^10^ | 5.4 × 10^7^ | 4.4 × 10^8^ | neg. | neg. | neg. | neg. |
| 26-Oct-2022 | 2.0 × 10^9^ | 5.3 × 10^10^ | 1.4 × 10^8^ | 2.8 × 10^9^ | neg. | neg. | 9.1 × 10^7^ | 2.5 × 10^7^ |
| 8-Nov-2022 | 4.7 × 10^9^ | 3.7 × 10^10^ | 8.7 × 10^8^ | 2.1 × 10^9^ | neg. | neg. | neg. | neg. |
| 9-Nov-2022 | 2.8 × 10^9^ | 1.1 × 10^11^ | 1.2 × 10^9^ | 6.1 × 10^10^ | neg. | neg. | 3.2 × 10^6^ | 6.3 × 10^6^ |
| 22-Nov-2022 | 1.2 × 10^9^ | 3.5 × 10^10^ | 9.7 × 10^7^ | 1.5 × 10^9^ | 1.6 × 10^6^ | neg. | neg. | neg. |
| 23-Nov-2022 | 1.6 × 10^9^ | 3.8 × 10^10^ | 2.6 × 10^8^ | 1.4 × 10^9^ | neg. | neg. | neg. | 7.9 × 10^7^ |
| 29-Nov-2022 | 1.8 × 10^9^ | 1.0 × 10^10^ | 5.3 × 10^8^ | 8.2 × 10^8^ | 1.3 × 10^6^ | neg. | 5.5 × 10^6^ | neg. |
| 30-Nov-2022 | 4.6 × 10^9^ | 3.4 × 10^10^ | 1.7 × 10^9^ | 2.8 × 10^9^ | neg. | neg. | neg. | neg. |
| 6-Dec-2022 | neg. | 3.5 × 10^10^ | n.t. | 6.8 × 10^8^ | n.t. | neg. | n.t. | neg. |
| 7-Dec-2022 | 2.2 × 10^9^ | 4.1 × 10^10^ | 2.3 × 10^8^ | 1.2 × 10^9^ | 1.9 × 10^7^ | neg. | neg. | neg. |
| 21-Dec-2022 | 1.0 × 10^9^ | 5.2 × 10^10^ | 8.5 × 10^7^ | 8.7 × 10^8^ | 1.8 × 10^7^ | neg. | neg. | neg. |

^a^ n.t. - not tested.

^b^ neg. - none detected.
